# The relationship between parental academic upward social comparison perception and short video addiction in junior high school students

**DOI:** 10.3389/fpsyt.2026.1953602

**Published:** 2026-09-23

**Authors:** Hang Lv, Zhouting Cai, Bin Liu, Chengbin Guan, Silu Shrestha, Yang Hao

**Affiliations:** 1 College of Education, Yangzhou University, Yangzhou, China; 2 Jiangsu Province Sihong Middle School, Suqian, China; 3 Affiliated Brain Hospital of Nanjing Medical University, Nanjing, China; 4 School of Sociology, Wuhan University, Wuhan, China

**Keywords:** adolescent mental health, fear of failure, negative core self-evaluation, parental academic upward social comparison perception, short video addiction

## Abstract

**Background:**

This study used a cross-sectional design and analyzed the relationship between parental academic upward social comparison perception (PAUSCP) and short video addiction (SVA) among junior high school students in Jiangsu Province, China, and explored the mediation of negative core self-evaluation (NCSE) and fear of failure (FOF).

**Methods:**

Using a cross-sectional research design, the study administered the parental academic upward social comparison perception scale, core self-evaluation scale, fear of failure scale and the short video addiction scale to 988 junior high school students from two schools in Jiangsu Province in April 2025 using multi-stage stratified cluster sampling to analyze chain mediation.

**Results:**

A total of 47.3% (n = 467) of the participants screened positive for SVA symptoms. The total indirect effect of PAUSCP on SVA through NCSE and FOF was significant (IE = 0.194, 95% CrI [0.158, 0.234]). Specifically, all three mediation pathways were significant: via NCSE alone (IE = 0.142, 95% CrI [0.112, 0.176]), via FOF alone (IE = 0.035, 95% CrI [0.017, 0.056]), and via the sequential chain pathway (IE = 0.017, 95% CrI [0.008, 0.027]).

**Conclusion:**

These findings suggest that higher PAUSCP is concurrently associated with higher SVA symptoms and that NCSE and FOF statistically account for part of this association in the specified model. PAUSCP was associated with SVA among the surveyed junior high school students through the statistical pathways involving NCSE and FOF. These findings may provide guidance for family-based interventions targeting short video addiction among junior high school students.

## 1 Introduction

Short video platforms have become one of the most widely used media among adolescents in recent years. Excessive use of short videos may lead to short video addiction (SVA), characterized by difficulties in controlling short-video use and persistent engagement despite adverse consequences (1). Short-video use may also function as an escapist or avoidant coping response, particularly when adolescents use such platforms to temporarily disengage from school-related stress or negative emotional experiences (2). International research indicates that internet and smartphone addiction exhibit high prevalence among adolescents and significantly worsen under stressful conditions, highlighting the critical role of emotion-regulation failure in the development of addictive behaviors (3, 4). Further meta-analytic evidence demonstrates that maladaptive emotion-regulation strategies—such as rumination and avoidance—significantly increase adolescents’ digital media addiction (5). Consequently, SVA can be conceptualized as a behavioral addiction characterized by the core mechanisms of emotional escape and regulatory imbalance (6). This issue is particularly pronounced among Chinese minors: the addiction rate among high school students is nearly twice that of junior high school students (7), underscoring the urgent need to investigate its psychosocial determinants.

Chinese adolescents face exceptionally intense academic pressure, which is deeply rooted in the country’s sociocultural context—academic achievement is a paramount concern for parents, and familial expectations regarding academic performance remain persistently high (8). Academic pressure has been shown to longitudinally predict depressive symptoms and self-harm behaviors persisting into early adulthood (9), underscoring the mental health burden faced by adolescents in high-competition educational contexts. A prominent source of this pressure is the practice of parents comparing their children’s academic performance to that of high-achieving peers. This phenomenon—whereby children perceive academic upward social comparisons made by their parents—is referred to as parental academic upward social comparison Perception (PAUSCP) (8). Although some parents believe that such comparisons can motivate their children, it remains an open question whether PAUSCP actually fosters competitive learning or inadvertently triggers psychological distress and negative behavioral outcomes.

According to Social Comparison Theory, individuals who engage in upward social comparisons use others as benchmarks for self-evaluation, typically resulting in diminished self-appraisal (10). Although upward social comparison is theoretically associated with motivational enhancement, empirical evidence indicates that it more often elicits feelings of inadequacy and negative self-evaluation (11). Pressure stemming from parents’ high academic expectations exerts a significant negative impact on adolescents’ mental health, and greater perceived academic stress is associated with more severe mental health problems (12). When such pressure goes unaddressed, adolescents frequently resort to instant entertainment media—such as short videos—as a temporary escape from academic burdens, thereby developing avoidance-based coping patterns that further exacerbate psychological distress (13). From a cognitive-behavioral perspective, the I-PACE model proposes that cognitive and affective responses interact in the development and maintenance of addictive behaviors (14). Both frameworks recognize cognition and emotion as critical mediators through which situational stressors are translated into dysfunctional behavioral outcomes. Taken together, these perspectives suggest that PAUSCP may be positively associated with SVA in adolescents.

Hypothesis 1: PAUSCP is significantly and positively correlated with SVA in junior high school students.

Existing evidence suggests that there may be a chain-like mediating mechanism between these two variables. Within this cognitive transmission pathway, Core self-evaluation (CSE) refers to individuals’ fundamental evaluations of themselves, encompassing self-esteem, generalized self-efficacy, emotional stability, and locus of control (15). In the present study, the construct is operationalized as negative core self-evaluation (NCSE) to capture adolescents’ maladaptive self-appraisals. Parental social comparison may be particularly relevant to adolescents’ self-perceptions: perceived parental social comparison has been associated with adolescents’ academic self-efficacy and self-handicapping (8), suggesting that repeated unfavorable comparisons made by parents may be associated with more negative self-appraisals in their children.

In the domain of digital media use, prior research has established that adolescents with higher NCSE are more likely to engage in problematic smartphone use (16). This finding is further corroborated by longitudinal evidence showing that negative self-evaluation is among the most influential nodes predicting problematic internet use over time (17). As one of the most immersive mobile media currently available, short videos—characterized by low cognitive demands and immediate gratification—serve as a highly accessible channel for behavioral avoidance among individuals with negative self-perceptions. Based on these findings, this study proposes:

Hypothesis 2: Negative core self-evaluation (NCSE) mediates the relationship between PAUSCP and SVA.

Beyond the cognitive evaluation pathway, emotional responses constitute an equally important mediating link in the transmission from PAUSCP to SVA. FOF involves negative appraisals of the anticipated consequences of failure, such as shame, devaluation of the self, or concern about disappointing significant others (18). Evidence from adolescent populations indicates that high educational expectations are associated with greater fear of failure and stress-related negative affect (19). In the context of parental academic comparison, being repeatedly confronted with higher-achieving peers may similarly make the possibility and perceived consequences of academic failure more salient. However, because direct evidence linking PAUSCP specifically to FOF remains limited, this pathway is proposed on theoretical grounds and tested empirically in the present study. At the behavioral level, within the framework of achievement motivation theory, FOF drives widespread and persistent avoidance behaviors through symbolic generalization mechanisms (20, 21). Problematic short video use is a typical example: short video content provides rich sensory stimulation with minimal cognitive effort, constituting a “low-effort, high-reward” exchange that continuously reinforces avoidance behavior (22, 23). When individuals turn to short videos to escape negative emotions, their usage patterns are significantly positively associated with anxiety and depression (24). Similarly, in academic contexts, individuals use mobile devices to temporarily relieve stress and regulate negative affect, thereby reinforcing problematic usage (25). Accordingly, this study proposes:

Hypothesis 3: FOF mediates the relationship between PAUSCP and SVA.

Previous studies have provided evidence for the individual associations among parental social comparison, negative self-evaluation, fear of failure, and problematic digital-media use. However, these constructs have rarely been integrated within a single model. The proposed PAUSCP–NCSE–FOF–SVA sequential pathway therefore represents a theoretically specified extension that is examined in the present study.

Based on the cognitive-affective processes described in the I-PACE model, this study further proposes a sequential indirect pathway linking PAUSCP, NCSE, FOF, and SVA (26). Negative self-evaluation may be associated with greater sensitivity to the anticipated consequences of failure among adolescents, particularly in evaluative academic contexts where parental expectations are salient. At the same time, higher FOF may also be accompanied by a stronger tendency to engage in short-video use, such as turning to immediately rewarding short-video activities to temporarily avoid distressing achievement-related experiences. In the present study, the I-PACE model serves as the overarching theoretical framework. PAUSCP is conceptualized as an external social-contextual input because it reflects adolescents’ perceived exposure to parental academic comparison in their immediate environment. Social comparison theory helps explain how such experiences may be associated with negative self-appraisal, whereas achievement-related theory helps explain how negative self-appraisal may be accompanied by fear of failure. Within this integrated framework, NCSE and FOF represent theoretically relevant cognitive and affective correlates linking PAUSCP with SVA.

Hypothesis 4: Negative core self-evaluation (NCSE) and FOF sequentially mediate the relationship between PAUSCP and SVA (PAUSCP → NCSE → FOF → SVA).

(Model Path: Theoretically specified statistical order: PAUSCP, NCSE, FOF, and SVA.). The hypothesized cross-sectional indirect-association model is presented in **Figure 1**.

**Figure 1:**
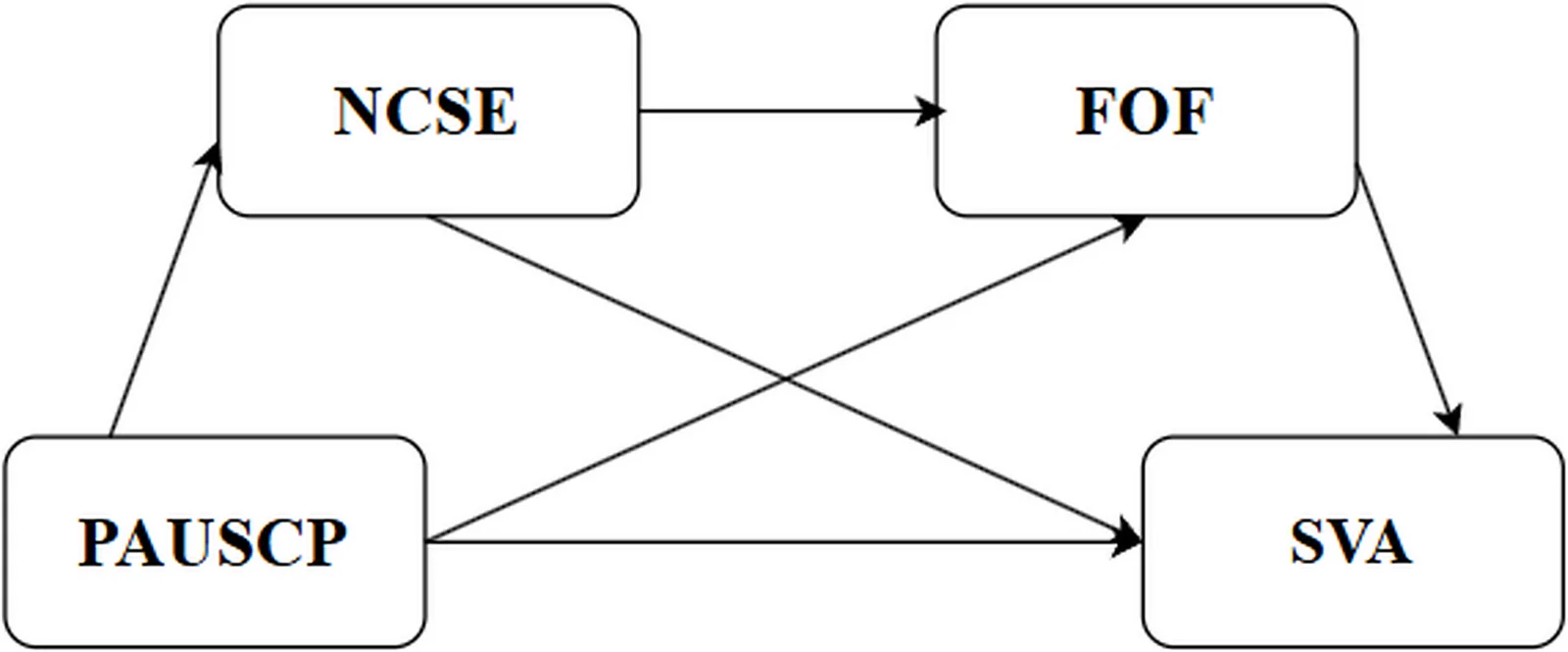
Hypothesized cross-sectional indirect-association model involving PAUSCP, NCSE, FOF, and SVA. PAUSCP, parental academic upward social comparison perception; NCSE, negative core self-evaluation; FOF, fear of failure; SVA, short video addiction.

## 2 Participants and methods

### 2.1 Procedure

The study was conducted in April 2025 after obtaining ethical approval from the Ethics Committee of the School of Education, Yangzhou University (YZU-JKY-2025040303). Prior to data collection, the researchers informed school administrators, students, and their parents or legal guardians about the study objectives and procedures. Written informed consent was obtained from the parents or legal guardians, and written assent was obtained from the adolescent participants before questionnaire completion. Questionnaires were administered in classroom settings using a paper-and-pencil format under the supervision of the researchers. Participants could withdraw at any time without penalty. Participation was voluntary, and students who chose not to participate could engage in their regular schoolwork during the activity period. Teachers were present only at the beginning of the session to provide general instructions and were not present during questionnaire completion. All questionnaires were completed anonymously, and no personally identifiable information was collected. Completing the questionnaires required approximately 15 minutes, after which all questionnaires were collected for data analysis. Regardless of whether they participated in the study, all students present received a small gift.

### 2.2 Participants

At the study-planning stage, reliable prior estimates of class-level intraclass correlation coefficients (ICCs) for the focal variables in this population were unavailable. Therefore, an empirically supported design effect could not be specified, and deff was conservatively set to 1 as a simplifying assumption for the initial sample-size calculation. Based on the standard sampling methodology for cross-sectional surveys, the sample size was calculated as follows: $n=deff\times \frac{{\mu^{2}}_{\alpha /2}\rho \left(1-\rho \right)}{\delta^{2}}$, where *α* = 0.05, *deff* = 1, *δ* = 0.2*ρ*, *ρ* = 30.13% (27). With a 10% inefficiency rate and stratified sampling across three junior high school grades (3 layers), a minimum of 743 participants was required. The anticipated screening-positive rate for short-video addiction symptoms of 30.13% was based on a previous survey of 604 Chinese adolescents aged 14–22 years, in which 182 participants screened positive for short-video addiction symptoms using the diagnostic items of the Short Video Addiction Scale (28). This estimate was selected because that study investigated the same outcome in a Chinese adolescent population and used the same short-video addiction instrument as the present study. To allow for an anticipated additional 10% nonresponse or incomplete-response rate, the required sample size was adjusted to 743/(1 − 0.10), resulting in a minimum target sample of 826. The data were collected in April 2025. The study employed a multi-stage stratified cluster sampling method. First, one selective and one non-selective junior high school were selected in Jiangsu province, China (first-level clusters). In this study, “selective” and “non-selective” corresponded to the locally recognized classification of key and non-key junior high schools, respectively. This was a school-level classification used in the sampling process. Second, all three grades were selected as second-level clusters. Finally, four classes were randomly sampled within each grade using stratified cluster sampling. A total of 1,074 questionnaires were distributed, and 988 valid responses were collected. Questionnaires were considered invalid primarily under the following circumstances: a substantial number of missing items or incomplete responses; obvious patterned or mechanical responding; or other data-quality problems that could be objectively identified from the original questionnaire records. The valid response rate was 92.0% (988/1,074). Among this finalized sample of 988 Chinese junior high school students (M = 13.12, SD = 1.16), 472 were male (47.8%) and 516 were female (52.2%). Regarding grade levels, 332 students (33.6%) were in the 7th grade, 337 (34.1%) in the 8th grade, and 319 (32.3%) in the 9th grade. In terms of family structure, 660 participants (66.8%) were from two-parent families, while 328 (33.2%) were from non-two-parent families. Additionally, 480 students (48.6%) attended selective school, and 508 (51.4%) attended non-selective school.

### 2.3 Measures

#### 2.3.1 Sociodemographic information

A self-administered demographic questionnaire was used to collect participants’ basic background information. This questionnaire assessed four primary sociodemographic factors: gender(female=1, male=2), grade level (7^th^ grade=1, 8^th^ grade=2, or 9th grade=3), school type (selective=1 or non-selective school=0), and family structure (two-parent family=1 or non-two-parent family=0).

#### 2.3.2 Parental academic upward social comparison perception

Parental academic upward social comparison perception, which evaluates adolescents’ perceptions of parents’ comparisons with higher-achieving peers, was assessed using the Parental Academic Upward Social Comparison Perception Scale revised by Xing et al. (8). It is a three-item unidimensional self-report measure evaluating adolescents’ perceptions of parents’ comparisons with higher-achieving peers (e.g., “When another student has a better school record or earns higher scores than I do, my father or mother feels frustrated with me.”). Items were rated on a 5-point Likert scale (1 = does not correspond at all to 5 = corresponds exactly), with higher scores indicating stronger perceived comparison. Previous studies reported satisfactory psychometric properties (8). In the present study, McDonald’s ω was 0.820, indicating acceptable reliability for adolescent samples.

#### 2.3.3 Core self-evaluation

Core self-evaluations were measured using the Core Self-Evaluation Scale (CSES) used by Hao et al. (29), which has demonstrated good reliability and validity among adolescents (30). This scale includes six negative core self-statement items that measure an individual’s negative core self-evaluation. The items reflect negative appraisals of one’s abilities, self-worth, and overall self-concept (e.g., “Sometimes I feel depressed”). The scale is a self-report measure, and all items are rated on a 5-point Likert scale (1 = strongly disagree to 5 = strongly agree), with higher scores indicating stronger negative core self-evaluation. In this study, McDonald’s ω was 0.877. Confirmatory factor analysis indicated an acceptable model fit. The six items were specified to load on a single latent factor, yielding χ^2^ = 52.091, df = 9, CFI = 0.984, TLI = 0.973, RMSEA = 0.070, and SRMR = 0.023. These results suggest that the scale demonstrated good construct validity.

#### 2.3.4 Fear of failure

Fear of failure, reflecting negative cognitive and emotional responses to actual or anticipated failure, was assessed using the five-item Fear of Failure Scale revised by Hao et al (31). An example item is “I am unable to get the support or help I need.” Responses are rated on a 5-point Likert scale (1 = not afraid at all to 5 = extremely afraid), with higher scores indicating greater fear of failure. The Chinese version used in the present study was previously examined primarily in a college-student population; therefore, evidence from that validation sample should not be assumed to establish its psychometric validity specifically among junior high school students. In the present sample, McDonald’s ω was 0.777, indicating acceptable internal consistency. Confirmatory factor analysis was conducted by specifying the five items to load on a single latent factor, yielding χ^2^ = 55.001, df = 5, CFI = 0.959, TLI = 0.918, RMSEA = 0.101, and SRMR = 0.037. These results suggest that the scale demonstrated good construct validity.

#### 2.3.5 Short video addiction

Short video addiction was assessed using the 14-item Short Video Addiction Scale originally developed by Qin et al. (32) for Chinese college students. The scale comprises four dimensions: loss of control, withdrawal reactions, avoidance behavior, and reduced efficiency. Items are rated on a 5-point Likert scale (1 = strongly disagree to 5 = strongly agree). An example item is: “You find that when you have to do other important things, you end up getting lost in watching short videos, and this has caused you some trouble”. The 14 items of the original scale included seven diagnostic screening items. Participants were classified as screening positive for SVA symptoms if at least four of the seven diagnostic screening items were rated 2 or higher. The scale has been examined for applicability among adolescents in Jiangsu Province, China, and has demonstrated good reliability and validity (33). In the present sample, the scale showed high internal consistency (McDonald’s ω = 0.935). Confirmatory factor analysis was conducted by specifying the 14 items to load on four latent factors, yielding χ^2^ = 232.010, df = 71, CFI = 0.979, TLI = 0.900, RMSEA = 0.048, and SRMR = 0.025. These results suggest that the scale demonstrated good construct validity.

### 2.4 Statistical methods

Bayesian mediation analyses were performed using JASP 0.97.1. Given the absence of clear parameter ranges in the existing literature for the intercepts, path coefficients, standard deviations, and correlation coefficients in the present model, the prior distributions followed the default settings in JASP: ν ~ Normal(μ, σ), with μ = 0 and σ = 32; β ~ Normal(μ, σ), with μ = 0 and σ = 10; ψ ~ Gamma(α, β), with α = 1.0 and β = 0.5; and ρ ~ Gamma(α, β), with α = 1 and β = 1. MCMC sampling was conducted using three parallel chains, with 2,500 burn-in iterations followed by 5,000 sampling iterations per chain. MCMC convergence was assessed using trace plots, R-hat, and effective sample size (ESS). All parameters showed satisfactory convergence, with R-hat values ranging from 1.000 to 1.002 and ESS values exceeding 1,000. Harman’s single-factor test was used to assess common method bias. All measurement items were entered into an exploratory factor analysis. Four factors with eigenvalues greater than 1 were extracted, and the first factor accounted for 28.46% of the total variance, which was below the commonly used 40% threshold, suggesting that serious common method bias was unlikely to substantially affect the results.

## 3 Results

### 3.1 Screening positivity for short video addiction symptoms

Based on the screening criterion of the Short Video Addiction Scale, participants were classified as screening positive for SVA symptoms if at least four of the seven diagnostic screening items were rated 2 or higher. Among the 988 junior high school students included in the analysis, 467 met the scale-based screening criterion, corresponding to a screening-positive rate of 47.3%. In addition to the screening-positive rate, the mean SVA score was 3.10 (SD = 1.03).

The screening-positive rate for SVA symptoms differed significantly by gender, grade, and family structure, whereas no significant difference was observed by school type (**Table 1**). Female students had a higher screening-positive rate than male students (p = 0.029). Screening positivity also differed significantly by grade (p = 0.004), with Bonferroni comparisons indicating a higher screening-positive rate among ninth-grade students than among seventh-grade students. And adolescents from non-two-parent families had a higher screening-positive rate than those from two-parent families (p< 0.001). No significant difference was observed between students from selective and non-selective schools (p = 0.534). The corresponding Cramér’s V values were 0.069 for gender, 0.105 for grade, 0.020 for school type, and 0.151 for family structure. These findings represent unadjusted descriptive group differences. Detailed results are presented in **Table 1**.

**Table 1:** Screening positivity for short video addiction symptoms by sociodemographic characteristics [n (%)].

Characteristics	Screening negative	Screening positive	χ^2^	Cramér’s V	P
Gender			4.760	0.069	0.029
Male	266(56.356)_a_	206(43.644)_a_			
Female	255(49.419)_b_	261(50.581)_b_			
Grade			10.955	0.105	0.004
7th Grade	196(59.036)_a_	136(40.964)_a_			
8th Grade	178(52.819)_a,b_	159(47.181)_a,b_			
9th Grade	147(46.082)_b_	172(53.918)_b_			
School Type			0.388	0.020	0.534
Non-Selective	263(51.772)_a_	245(48.228)_a_			
Selective	258(53.750)_a_	222(46.250)_a_			
Family Structure			22.384	0.151	<0.001
Non-two-parent	138(42.073)_a_	190(57.927)_a_			
Two-parent	383(58.030)_b_	277(41.970)_b_			

a, b indicate Bonferroni comparisons (*α* = 0.05).

### 3.2 Correlation analysis

PAUSCP was positively correlated with NCSE (r = 0.443), FOF (r = 0.445), and SVA (r = 0.465). In addition, both NCSE (r = 0.503) and FOF (r = 0.394) were positively correlated with SVA. All correlations were statistically significant at P< 0.01.

### 3.3 Mediation analysis

Gender, grade, two-parent family, and selective school were included as covariates in all models. A Bayesian mediation approach was employed using JASP (version 0.97.1), with weakly informative priors and Markov Chain Monte Carlo (MCMC) sampling (burn-in = 2,500; sampling iterations = 5,000). Model convergence was confirmed by R-hat< 1.01 and ESS > 1,000 for all parameters.

In the simple mediation model with NCSE as mediator (**Table 2**; **Figure 2**), model fit was acceptable (PPP = 0.499). The model yielded a positive estimated direct association between PAUSCP and SVA (DE = 0.305 [95% credible interval (CrI): 0.248, 0.363]), a total effect (TE = 0.465 [95% CrI: 0.409, 0.519]), and a significant indirect effect via NCSE (IE = 0.159 [95% CrI: 0.128, 0.193]), with the proportion mediated estimated at 34.19%.

**Table 2:** Path coefficients (Simple Mediation Model: NCSE as Mediator).

Path	Posterior			95% Credible Interval		R-hat	ESS (bulk)	ESS (tail)
	Mean	Median	SD	Lower	Upper			
PAUSCP→SVA	0.305	0.305	0.029	0.248	0.363	1.000	28879	12375
PAUSCP→NCSE	0.409	0.409	0.027	0.357	0.461	1.000	36004	10654
NCSE→SVA	0.389	0.389	0.032	0.327	0.452	1.000	27446	12990
Covariates								
Gender →SVA	0.119	0.119	0.053	0.015	0.221	1.000	39247	9906
Grade →SVA	0.096	0.096	0.033	0.030	0.161	1.000	37028	10752
Selective school →SVA	-0.067	-0.068	0.052	-0.169	0.034	1.001	36070	11001
Two-parent family →SVA	-0.267	-0.266	0.056	-0.376	-0.157	1.000	35339	10913
Gender →NCSE	0.008	0.008	0.054	-0.096	0.114	1.001	37750	10324
Grade →NCSE	0.052	0.052	0.032	-0.012	0.115	1.000	33776	9681
Selective school →NCSE	-0.102	-0.102	0.053	-0.204	0.002	1.000	37113	10653
Two-parent family →NCSE	-0.118	-0.119	0.058	-0.230	-0.006	1.000	34403	10810

All reported coefficients are unstandardized.

**Figure 2:**
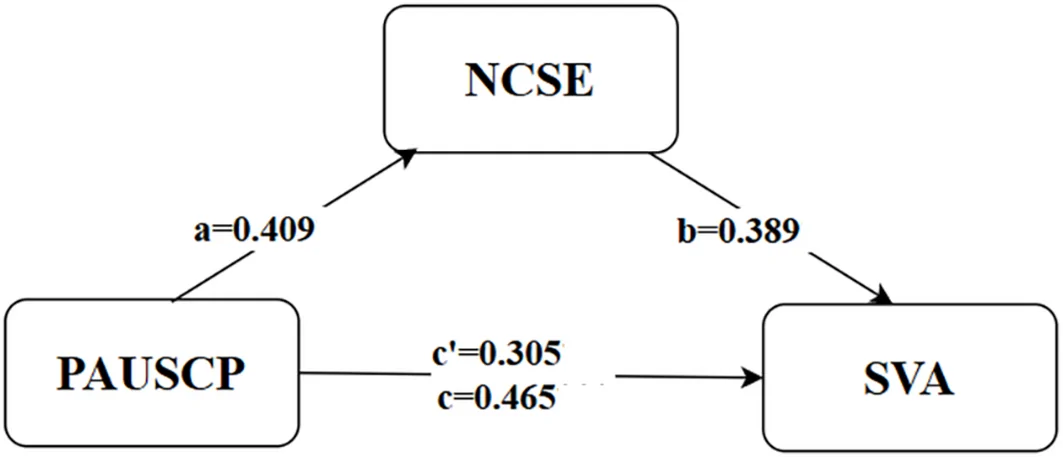
Simple mediation model of PAUSCP on SVA via NCSE. Values represent posterior medians of unstandardized path coefficients with 95% credible intervals (95% CrIs). Gender, grade, selective school, and two-parent family were included as covariates in the model but are not shown in the figure for clarity.

In the simple mediation model with FOF as mediator (**Table 3**; **Figure 3**), model fit was acceptable (PPP = 0.502). Results indicated a significant direct effect of PAUSCP on SVA (DE = 0.366 [95% CrI: 0.307, 0.428]), a total effect (TE = 0.464 [95% CrI: 0.409, 0.521]), and a significant indirect effect via FOF (IE = 0.098 [95% CrI: 0.070, 0.128]), with the proportion mediated PM = 21.12%.

**Table 3:** Path coefficients (Simple Mediation Model: FOF as Mediator).

Path	Posterior			95% Credible Interval		R-hat	ESS(bulk)	ESS(tail)
	Mean	Median	SD	Lower	Upper			
PAUSCP→SVA	0.366	0.366	0.031	0.307	0.428	1.000	19981	12693
PAUSCP→FOF	0.364	0.364	0.024	0.317	0.410	1.000	26814	11077
FOF→SVA	0.271	0.271	0.037	0.197	0.344	1.000	20078	12464
Covariates								
Gender →SVA	0.117	0.116	0.056	0.006	0.226	1.000	22828	11654
Grade →SVA	0.096	0.096	0.034	0.030	0.163	1.000	24463	11515
Selective school →SVA	-0.091	-0.091	0.055	-0.198	0.018	1.000	26644	12087
Two-parent family →SVA	-0.299	-0.298	0.060	-0.417	-0.180	1.000	23926	11182
Gender →FOF	0.023	0.023	0.047	-0.069	0.115	1.000	25466	11117
Grade →FOF	0.074	0.074	0.029	0.017	0.131	1.000	26459	11438
Selective school →FOF	-0.058	-0.057	0.047	-0.151	0.035	1.000	27665	11486
Two-parent family →FOF	-0.052	-0.052	0.051	-0.151	0.048	1.000	25790	10954

All reported coefficients are unstandardized.

**Figure 3:**
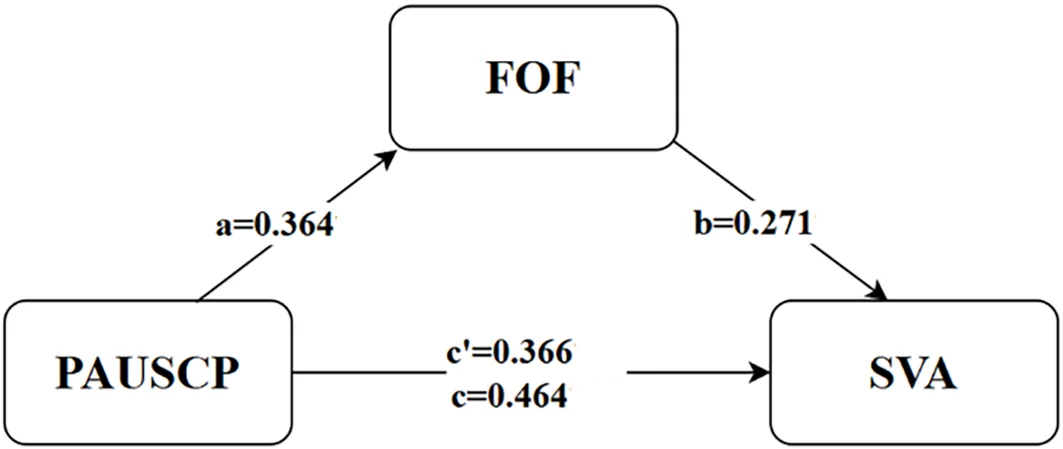
Simple Mediation Model of PAUSCP on SVA via FOF. Values represent posterior medians of unstandardized path coefficients with 95% credible intervals (95% CrIs). Gender, grade, selective school, and two-parent family were included as covariates in the model but are not shown in the figure for clarity.

In the chain mediation model (**Tables 4**, **5**; **Figure 4**), model fit was acceptable (PPP = 0.504). Results showed a direct effect of PAUSCP on SVA (DE = 0.270 [95% CrI: 0.209, 0.330]), a total effect (TE = 0.465 [95% CrI: 0.409, 0.520]), and a significant total indirect effect (TIE = 0.194 [95% CrI: 0.158, 0.234]), with PM = 41.94% (34). The indirect effect via NCSE alone was IE = 0.142 [95% CrI: 0.112, 0.176]; via FOF alone was IE = 0.035 [95% CrI: 0.017, 0.056]; via the chain pathway PAUSCP→NCSE→FOF→SVA was IE = 0.017 [95% CrI: 0.008, 0.027]. Effect decomposition is presented in **Table 5**. Among the three indirect pathways, the pathway through NCSE alone accounted for the largest proportion of the total indirect effect (73.20%), followed by the FOF pathway (18.04%) and the chain pathway (8.76%).

**Table 4:** Path coefficients (Chain mediation model).

Path	Posterior			95% Credible Interval		R-hat	ESS(bulk)	ESS(tail)
	Mean	Median	SD	Lower	Upper			
PAUSCP→SVA	0.270	0.270	0.031	0.209	0.330	1.000	18544	11812
NCSE→SVA	0.348	0.348	0.033	0.283	0.413	1.000	19234	12754
FOF→SVA	0.145	0.145	0.038	0.071	0.218	1.000	19183	12122
PAUSCP→NCSE	0.409	0.409	0.027	0.356	0.461	1.001	23447	11630
PAUSCP→FOF	0.247	0.247	0.025	0.198	0.295	1.000	19633	11671
NCSE→FOF	0.286	0.285	0.027	0.233	0.338	1.000	19053	12732
Covariates								
Gender →SVA	0.117	0.117	0.052	0.015	0.221	1.000	24376	11383
Grade →SVA	0.087	0.087	0.033	0.023	0.152	1.000	25364	11516
Selective school →SVA	-0.063	-0.063	0.053	-0.165	0.040	1.000	24370	10686
Two-parent family →SVA	-0.264	-0.264	0.056	-0.372	-0.153	1.001	26440	11203
Gender →NCSE	0.008	0.008	0.053	-0.098	0.112	1.001	23511	11146
Grade →NCSE	0.052	0.052	0.033	-0.012	0.116	1.001	23735	11193
Selective school →NCSE	-0.102	-0.103	0.054	-0.206	0.002	1.000	24783	12114
Two-parent family →NCSE	-0.119	-0.119	0.057	-0.229	-0.008	1.000	25455	11006
Gender →FOF	0.020	0.021	0.045	-0.067	0.108	1.000	25626	10644
Grade →FOF	0.059	0.059	0.027	0.006	0.113	1.000	23968	10748
Selective school →FOF	-0.029	-0.029	0.045	-0.117	0.059	1.000	25797	11062
Two-parent family →FOF	-0.018	-0.018	0.047	-0.110	0.074	1.000	24465	11755

All reported coefficients are unstandardized.

**Table 5:** Effect decomposition.

Path	Posterior			95% Credible Interval		R-hat	ESS(bulk)	ESS(tail)
	Mean	Median	SD	Lower	Upper			
Mediation model of NCSE								
PAUSCP→SVA, Total effect	0.464	0.465	0.028	0.409	0.519	1.000	30641	12385
PAUSCP→SVA, Direct effect	0.305	0.305	0.029	0.248	0.363	1.000	28879	12375
PAUSCP→NCSE→SVA	0.159	0.159	0.017	0.128	0.193	1.000	29425	11549
Mediation model of FOF								
PAUSCP→SVA, Total effect	0.465	0.464	0.028	0.409	0.521	1.000	28360	13185
PAUSCP→SVA, Direct effect	0.366	0.366	0.031	0.307	0.428	1.000	19981	12693
PAUSCP→FOF→SVA	0.099	0.098	0.015	0.070	0.128	1.000	20819	12641
Chain mediation model								
PAUSCP→SVA, Total effect	0.465	0.465	0.028	0.409	0.520	1.000	24310	13081
PAUSCP→SVA, Direct effect	0.270	0.270	0.031	0.209	0.330	1.000	18544	11812
PAUSCP→SVA, Total indirect effect	0.195	0.194	0.019	0.158	0.234	1.000	21122	12200
PAUSCP→NCSE→SVA	0.142	0.142	0.017	0.112	0.176	1.000	20045	12114
PAUSCP→FOF→SVA	0.036	0.035	0.010	0.017	0.056	1.000	19806	12370
PAUSCP→NCSE→FOF→SVA	0.017	0.017	0.005	0.008	0.027	1.000	19070	12359

All reported effect estimates are unstandardized.

**Figure 4:**
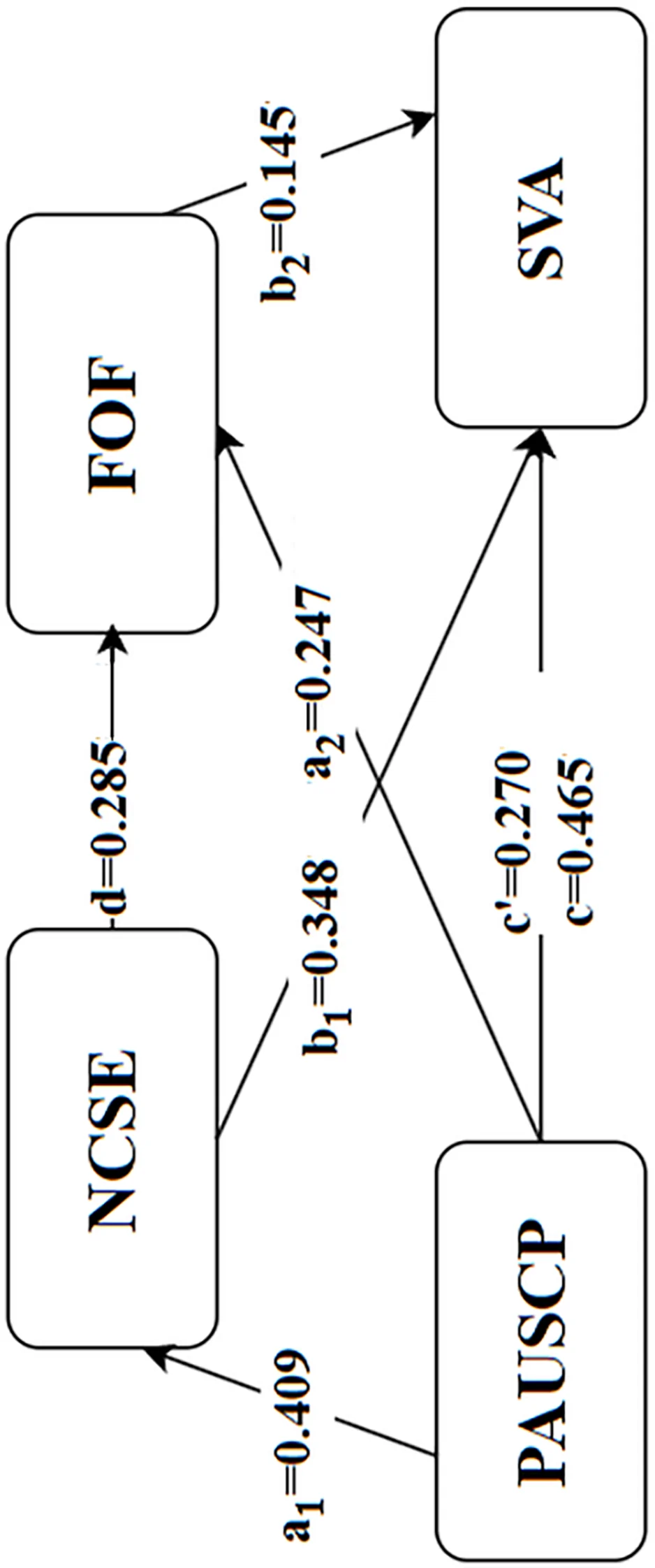
Chain mediation model of PAUSCP on SVA via NCSE and FOF. Values represent posterior medians of unstandardized path coefficients with 95% credible intervals (95% CrIs). Gender, grade, selective school, and two-parent family were included as covariates in the model but are not shown in the figure for clarity.

## 4 Discussion

The present study found that 47.3% of the surveyed junior high school students from two schools in Jiangsu Province screened positive for SVA symptoms. This relatively high proportion of screening-positive results suggests that SVA symptoms may be relatively common in the surveyed population. Gender, grade, and family structure were significantly associated with screening positivity for SVA symptoms: female students, ninth-grade students, and adolescents from non-two-parent families showed higher proportions of screening-positive results than male students, seventh-grade students, and adolescents from two-parent families, respectively. Previous adolescent research suggests that psychological processes associated with problematic smartphone or short-video use may differ by gender (35, 36); however, these findings do not establish why female students in the present sample showed a higher screening-positive rate. Similarly, the higher screening-positive rate among ninth-grade students may reflect developmental (37), academic, or social-contextual differences, whereas the pattern observed among adolescents from non-two-parent families may be associated with differences in family resources, parental supervision, or other unmeasured family factors.

### 4.1 Relationship between PAUSCP and SVA

The data analysis revealed a positive correlation between PAUSCP and SVA in junior high school students. Previous research has primarily focused on explicitly negative forms of parent-child interaction, such as parental abuse (38), neglect (39), and phubbing (40), all of which are positively correlated with adolescent internet or smartphone addiction. However, this study found that parental upward academic comparison, a form of Chinese parenting often intended as “positive encouragement,” was also associated with higher levels of SVA. According to classic Social Comparison Theory, upward comparison may trigger competitive behavior (10). Parents may adopt this approach in an effort to motivate their children’s academic competitiveness. However, the findings of this study indicate an unintended pattern: PAUSCP is correlated with higher levels of SVA, which contradicts parents’ intended motivational goals. Previous studies have also found that higher levels of SVA are negatively correlated with academic engagement (41). When adolescents perceive academic expectations from parents as harmful, it may not lead to increased academic motivation. Instead, it may increase the likelihood of avoidance behaviors and internet addiction (42). Therefore, in the context of adolescent addictive behaviors, the I-PACE model may explain the relationship between parents’ upward academic comparisons and short video addiction among Chinese junior high school students more appropriately than Social Comparison Theory. This study extends previous research in three specific ways. First, whereas studies of academic pressure have generally examined broad academic demands or parental expectations, the present study focuses specifically on adolescents’ perceptions of parental academic upward social comparison as a culturally relevant form of family academic communication. Second, unlike research centered on general internet or smartphone addiction, this study examines addiction symptoms related specifically to short-video use. Third, previous studies of parent–child interaction and addictive behaviors have primarily emphasized explicitly adverse experiences, such as abuse, neglect, and parental phubbing, whereas the present study considers a parenting practice that may be intended as motivational but may nevertheless be associated with maladaptive outcomes.

### 4.2 Chain mediation pathway

The specified model yielded a sequential indirect statistical association involving PAUSCP, NCSE, FOF, and SVA (26). Prior studies have also found that negative emotional states such as anxiety, depression, and loneliness are reciprocally related to avoidance behaviors, including internet addiction (43). From a cultural-psychological perspective, emotional expression is often shaped by contextual and relational norms rather than being uniformly suppressed; for instance, collectivistic cultural contexts may encourage more situational or relationally-attuned emotional regulation compared with individualistic contexts (44). Cultural display rules can similarly modulate how and when emotions are expressed depending on social context (45). Within Chinese family contexts, parental comparative remarks intended as indirect encouragement may nonetheless be interpreted by adolescents as expressions of disappointment, illustrating how culturally-embedded communication styles can shape adolescents’ emotional experiences. For example, the common phrase “Look at how much better other children are than you” may be intended by parents as an indirect form of expectation and encouragement. However, from the adolescent’s perspective, this message is often perceived more directly as a sign of parental dissatisfaction. This perceived dissatisfaction may lower adolescents’ core self-evaluations and elicit negative emotional responses. To cope with these adverse experiences, adolescents may redirect their limited cognitive resources toward self-protective strategies, such as excessive short video browsing or other avoidant addictive behaviors, instead of engaging in academic efforts (46). The I-PACE model suggests that different types of addictive behaviors vary in their psychological mechanisms, particularly regarding the interplay of situational, cognitive, and emotional factors (26).

### 4.3 Simple mediation pathway via NCSE

The indirect association through NCSE was significant in the simple mediation model. In the chain mediation model, the NCSE-only indirect association was also significant. This pattern is consistent with prior research showing that negative core self-evaluation is positively associated with addictive behaviors (47, 48), including internet and smartphone addiction (16). The prominence of the NCSE-only pathway suggests that NCSE may play an important statistical role in accounting for part of the association between PAUSCP and SVA.

### 4.4 Simple mediation pathway via FOF

The positive association between fear of failure and short video addiction symptoms is broadly consistent with previous research linking failure-related concerns to avoidant coping behaviors. The model showed a significant indirect association between PAUSCP and SVA through FOF alone. Given that few previous studies have directly examined the association between FOF and SVA, the present findings provide important additional evidence. Specifically, parental comparison may be associated with greater anticipatory fear of failure, which may, in turn, be accompanied by increased reliance on short-video use as an avoidant coping strategy (21, 26).

### 4.5 Highlights

The present study extends previous research on academic pressure, smartphone addiction, and adverse parent–child interactions by examining parental academic upward social comparison as a culturally relevant family correlate of short video addiction symptoms. It further identifies NCSE and FOF as potentially relevant psychological processes within the I-PACE framework, while treating the observed indirect pathways as statistical rather than causal. These contributions provide a basis for further examination using longitudinal, multi-informant, and intervention designs.

### 4.6 Practical implications

In conclusion, against the backdrop of a rising prevalence of psychological problems among adolescents (49), parental upward academic comparison may not always achieve its intended motivational effects and may instead be associated with adverse outcomes. Related studies have also shown that upward social comparison is negatively associated with adolescents’ self-perceived competence and psychological well-being (50, 51). At a broader societal level, upward comparison has also been linked to increased perceptions of “involution” and excessive competitive behaviors (52). In the contemporary Chinese sociocultural context, *neijuan*, commonly translated as “involution,” refers to an escalating and self-perpetuating form of competition in which individuals continually invest greater effort and resources merely to maintain their relative position without obtaining proportionate benefits. It is characterized by perceived resource scarcity, competitive social norms, psychological pressure, and intensified competitive behavior. Unlike ordinary competition, *neijuan* also implies a sense of involuntary participation: individuals may feel compelled to continue competing because withdrawing could place them at a relative disadvantage, even when further effort produces diminishing returns (53). Given the current societal context characterized by involution, junior high school students may already be exposed to substantial social comparison and pressure in daily life (53). The present findings therefore suggest that parental upward academic comparison and adolescents’ interpretations of such comparisons may represent potentially relevant targets for future family-based prevention research. Future studies could examine whether reducing potentially stressful academic comparisons and promoting more supportive parent-child interactions are associated with better adolescent outcomes. In this context, greater attention to adolescents’ emotional well-being alongside their cognitive development may also warrant consideration in future intervention research (54). Similarly, fostering a positive self-concept and reducing negative self-evaluations may represent additional candidate targets for prevention programs (55). However, because the present study was cross-sectional and did not evaluate any intervention, the effectiveness of these approaches in reducing SVA symptoms remains to be established through longitudinal, experimental, and intervention studies.

### 4.7 Limitations and future directions

Several limitations should be noted. First, given the cross-sectional design of the present study, the temporal ordering and causal direction among the variables cannot be established. In addition to the theoretically proposed pathway, alternative explanations remain plausible. For example, SVA may be associated with poorer self-evaluation, greater academic difficulties, and heightened fear of failure, while unmeasured factors such as psychological distress may simultaneously influence both PAUSCP and SVA. Moreover, the associations among NCSE, FOF, and SVA may be reverse or reciprocal in nature. These alternative explanations should therefore be further examined using longitudinal or experimental designs.

In addition, the present study focused exclusively on junior high school students in Jiangsu province; future research should consider younger age groups, such as children in earlier developmental stages. Moreover, since SVA was assessed through self-report, future studies should integrate clinical psychiatric evaluations to improve diagnostic accuracy. In addition, class identifiers were not retained in the analytic dataset, which prevented us from estimating class-level intraclass correlations or conducting clustering-adjusted analyses; therefore, potential non-independence of observations arising from class-based cluster sampling could not be accounted for. Finally, the generalizability of the findings is limited due to the restricted sampling scope. Expanding the sample size and diversity could enhance the ecological validity of the results.

A further limitation concerns measurement: the FOF scale used in this study was originally validated primarily among college-student populations, and its psychometric properties in early adolescent samples have not been independently confirmed. Although acceptable reliability was observed in the present sample, recently developed age-specific instruments could be considered in future work, such as the fear-of-failure scale validated for adolescents aged 11–14 years (56) to further verify and extend the present findings.

## 5 Conclusion

PAUSCP was positively associated with SVA symptoms among junior high school students in Jiangsu province. The present study suggests that PAUSCP, NCSE, and FOF may be relevant variables for future longitudinal and intervention research concerning adolescent SVA.

## Data Availability

The raw data supporting the conclusions of this article will be made available by the authors, without undue reservation.
